# Analog programing of conducting-polymer dendritic interconnections and control of their morphology

**DOI:** 10.1038/s41467-021-27274-9

**Published:** 2021-11-25

**Authors:** Kamila Janzakova, Ankush Kumar, Mahdi Ghazal, Anna Susloparova, Yannick Coffinier, Fabien Alibart, Sébastien Pecqueur

**Affiliations:** 1 Univ. Lille, CNRS, Centrale Lille, Univ. Polytechnique Hauts-de-France, UMR 8520—IEMN, 59000 Lille, France; 2 Laboratoire Nanotechnologies & Nanosystèmes (LN2), CNRS, Université de Sherbrooke, Sherbrooke, QC J1X0A5 Canada

**Keywords:** Electrochemistry, Electrical and electronic engineering, Electronic devices

## Abstract

Although materials and processes are different from biological cells’, brain mimicries led to tremendous achievements in parallel information processing via neuromorphic engineering. Inexistent in electronics, we emulate dendritic morphogenesis by electropolymerization in water, aiming in operando material modification for hardware learning. Systematic study of applied voltage-pulse parameters details on tuning independently morphological aspects of micrometric dendrites’: fractal number, branching degree, asymmetry, density or length. Growths time-lapse image processing shows spatial features to be dynamically dependent, and expand distinctively before and after conductive bridging with two electro-generated dendrites. Circuit-element analysis and impedance spectroscopy confirms their morphological control in temporal windows where growth kinetics is finely perturbed by the input frequency and duty cycle. By the emulation of one’s most preponderant mechanisms for brain’s long-term memory, its implementation in vicinity of sensing arrays, neural probes or biochips shall greatly optimize computational costs and recognition required to classify high-dimensional patterns from complex environments.

## Abstract (web summary)

Despite advances in brain-inspired computing, existing electronics use top-down processes that do not compare with neural connections in the brain. Here, the authors report an electrically-tunable electropolymerization process that emulates and controls neural dendritic morphogenesis.

## Introduction

Brain-inspired computing has reached important milestones in the last decades, positioning it as a realistic alternative to conventional computers’ in the big data era^1^. Conventional computers’ limitations are both at the architectural level through the Von Neumann bottleneck and at the computational level through the deterministic nature of Boolean logic. As hardware, information processing technologies are intrinsically bound to materials that are chosen to carry quanta of information, and fabrication processes to shape them into dense structures of computing device elements^2–8^. In software, Machine-Learning based Artificial Neural Networks (ANN) are algorithms that have demonstrated above human-level performances for a large variety of high-dimensional data processing tasks such as image^9^, or speech recognition^10,11^. These achievements are currently enabled by the level of performances provided by modern computers that allow processing ANN models over realistic durations for their practical application. However, running such algorithms on current computers is highly energy-demanding comparatively to the brain, up to major concerns for applications as embedded computing for robotics or sensing for brain–machine interfacing. If important improvements at the algorithmic level certainly have to be done to reach brain-like performances in terms of power consumption, the hardware that supports such unconventional computing remains an open field for innovation in that sense^12^. One particular aspect that conventional electronics do not exploit is in operando device bottom-up engineering. On the contrary, such equivalent processes naturally occur in the brain in the form of diverse biogeneses that create physical connections, defining optimal computing topologies or network self-healing. In particular, brain’s dendrite morphogenesis originates on each neuron upon the whole life of a learning system, and controls each neuron’s growth as a full functionality for the brain’s long-term memory^13^. In conventional hardware, devices are manufactured in a top-down fashion prior to their use, and all possible electrically-erasable connections and network parameters need to be defined a priori. While the biological approach maximizes resources’ utilization, the electrical one requires oversizing network dimensions. Exploring the possibility of its embedding in electronic devices would provide disruptive solutions and perspectives for hardware ANN implementation.

To this end, this study focuses on electropolymerization to form electrical connections between nodes, at a surprising mimicry level with biological dendrites, with enough versatility to tune their morphology by the input voltage-pulse dynamics that activates the hard wiring. Neural dendritic patterns in the brain can be classified into thirteen families of three groups of dendritic fields^14^, and each family of dendritic arbors has specific characteristics of branching number, number of segmentation per dendrites, and the length and volume of the different branches and segments^15^. As dendrites’ morphology contributes to the electrophysiological features that yield their signaling^16^, the control of this versatility is essential to tune the signal propagation associated with the temporal characteristics of their memory property. Our study investigates how electrical events influence the growth of conducting dendrites and on how to changes the shape of such interconnections.

## Results

**Fig. 1:**
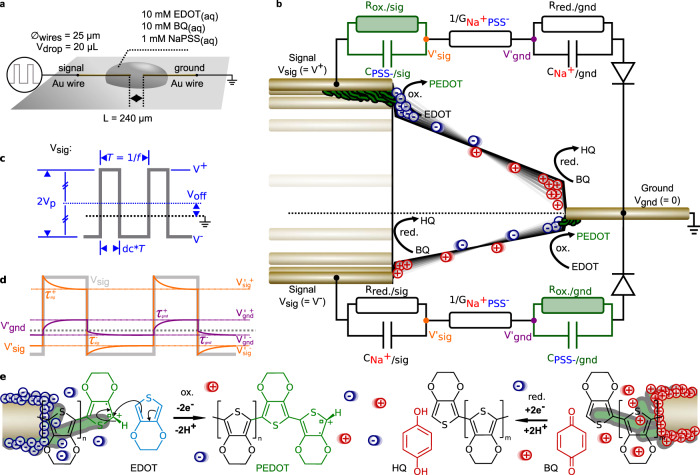
Working principle for the growth of conducting-polymer dendrites under pulse-voltage polarization. **a** Experimental setup to grow dendritic PEDOT on free-standing gold (Au) wires of 25 μm diameter, separated by a gap of *L* = 240 μm, polarized under square voltage on the signal wire, and the other wire grounded, in an aqueous electrolyte containing 10 mM of EDOT, 10 mM of BQ and 1 mM of NaPSS, as a *V*_drop_ = 20 μL drop. **b** Generic equivalent circuit of the electrochemical system, depicting the dynamics of the bias voltage across the gold wires, that yield to various potential dependent kinetics for the ion drifts thanks to the different RC circuit elements involved in the electrochemical system. **c** Definition of the *V*_sig_ pulse-voltage parameters investigated in this study, in terms of peak voltage amplitude *V*_p_, voltage offset *V*_off_, frequency *f*, duty cycle dc, anodic polarization *V*^+^, cathodic polarization *V*^−^ and period *T*. **d** Voltage diagram displaying the ion dynamics, inducing a time-dependent voltage-drop across the electrolyte, that results in different growth modes for the electropolymerized PEDOT dendrites. **e** Faradaic (redox) and Non-Faradaic (capacitive) processes occurring at both anodic and cathodic dendritic electrodes upon *V*_sig_ polarization.

Earlier results on 3,4-ethylenedioxythiophene (EDOT) bipolar electropolymerization showed the oxidative formation of millimeter-scaled PEDOT (ePEDOT) dendritic structures, supported by benzoquinone (BQ) reducing into hydroquinone (HQ)^17–22^. Advantages of such conductive bridging of polarizable nodes in a common electrolyte are local activation (individual programing), dependency on electric field’s direction (layer-to-layer interconnection parallelism), on field’s magnitude (spatial convolution), and voltage non-linearity for electro-activated polymerizations (neural rectification). Here, we study such process on free-standing gold wires in water (Fig. 1a). In such setup, process’ field-dependency is confirmed as the wires’ vicinity greatly conditions the dendrite formation at the same applied voltage. Water was used here as a singly electrolyte-supporting solvent^23^. Despite larger voltage biases than water’s electrochemical window, water splitting was never observed during operation as bubbling on wires was absent (except intentionally for the later study on the duty cycle). EDOT_(aq)_ and BQ_(aq)_ were always introduced equimolar at 10 mM, limited by the moderate solubility of EDOT in water^23^. Here, growths were performed by applying voltage pulses at the signal wire and grounding the other one, such that the applied voltage equals (Fig. 1c):

$${{{V}}}_{{{{{{\rm{sig}}}}}}}\left(t\right)-{{{V}}}_{{{{{{\rm{gnd}}}}}}}=\left\{\begin{array}{c}{{{V}}}^{+}\;{{{{{\rm{for}}}}}}\;t\in \left[\right.0{{{{{\rm{;dc}}}}}}* T\left[\right.{{{{{\rm{mod}}}}}}(T)\\ {{{V}}}^{-}\;{{{{{\rm{for}}}}}}\;t\in \left[\right.{{{{{\rm{dc}}}}}}* T{{{{{\rm{;}}}}}}T\left[\right.{{{{{\rm{mod}}}}}}(T)\end{array}\right.$$

With *V*^+^ = *V*_off_ + *V*_p_, the signal’s anodic polarization (*V*_p_ defined as peak voltage amplitude and *V*_off_ the voltage offset) applied for each period *T* = 1/*f* between 0 and dc*T (dc being the duty cycle) and *V*^−^ = *V*_off_ − *V*_p_, the signal’s cathodic polarization, applied for each period *T* between dc*T and *T*. As the setup is not electrochemically-potentiostated, voltage drops between both interfaces from *V*’_sig_ at the signal’s dendrite and *V*’_gnd_ at the ground one, as depicted in orange and purple in Fig. 1b. NaPSS_(aq)_ was chosen as an electrolyte to generate a highly doped ePEDOT dendrite of conductivity up to 1 kS/cm^24^. Considering Faradaic processes at both wire/electrolyte interfaces, the dynamics obey an idealized equivalent circuit depicted in Fig. 1b (assuming no mass-transfer limiting electrochemical impedances)^25^. In this model, the electrolytic conductance G_Na+PSS−_ accounts for both Na^+^_(aq)_ and PSS^−^_(aq)_ drift currents. Both ions accumulate at interfaces of the opposite polarity to form a dense cathodic PSS^−^_(aq)_ double-layer (capacitance *C*_PSS−_) and Na^+^_(aq)_ at the anode (*C*_Na+_). Both double layers permeation to neutral solutes rely on their packing, modulating the Faradaic processes of EDOT oxidation (resistance *R*_ox_) and BQ reduction (*R*_red_). Periodically, processes are reversed between both signal and grounded electrodes. Idealizing ionic charges/discharges to ohmic resistors/dielectric capacitors relaxations, surface potentials’ dynamics are depicted in Fig. 1d. We iteratively studied the influence of all four parameters, *V*_p_, *V*_off_, *f*, and dc that universally defined any pulse waveform, before exploiting his process for hardware event-based programming.

### Morphological influence with the peak voltage amplitude

**Fig. 2:**
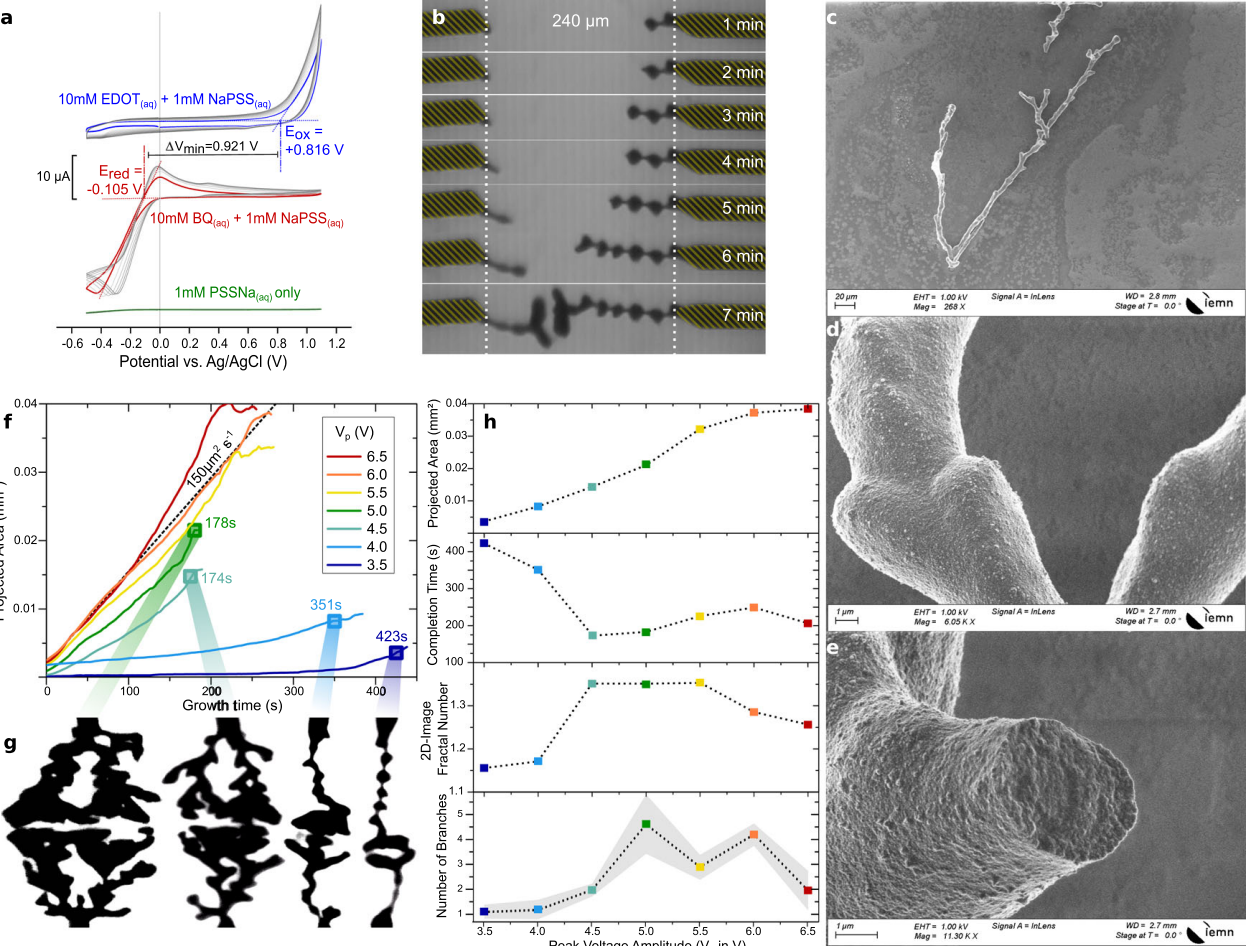
ePEDOT dendrites and their morphological control by the voltage amplitude (*V*_p_). **a** Cyclic Voltammetry for the two Au-wire 1 mM NaPSS_(aq)_ electrochemical system and its behavior without electrochemically active species (in green), with 10 mM EDOT_(aq)_ (in blue), and with 10 mM BQ_(aq)_ (in red). **b** Microscope pictures of one ePEDOT dendritic formation at different time lapses of electropolymerization (*V*_p_= 3.5 V, *V*_off_ = 0 V, *f* = 80 Hz, dc = 50%). The yellow stripped regions represent the position of the two gold wires (identified from their position at *t* = 0 s), in a fresh 10 mM EDOT_(aq)_ + 10 mM BQ_(aq)_ + 1 mM NaPSS_(aq)_ electrolyte. **c**–**e** Scanning electron microscope images of an ePEDOT dendritic structure after extraction from its electrolyte. **f** Projected area over time for different dendrites grown at different *V*_p_ (*V*_off_ = 0 V, *f* = 80 Hz, dc = 50%). **g** Contrasted microscope images of four different dendrites, grown at different *V*_p_ (*V*_off_ = 0 V, *f* = 80 Hz, dc = 50%), taken at their completion times (highlighted with square marks in **f**). **h** Relationship between *V*_p_ and projected area, completion time, their image’s fractal number, and the averaged value of apparent number of branches for dendrites grown up to completion time (*V*_off_ = 0 V, *f* = 80 Hz, dc = 50%). The grey_colored error interval on the number of branches is set to the mean value ± SD (six values along *L* for each *V*_p_). Scatters and curves’ color encodings with peak voltage amplitude in **f** and **h** are identical.

Cyclic voltammetry (CV) of the aforementioned setup shows that a minimum Δ*V*_min_ = 0.9 V is required to electropolymerize EDOT_(aq)_ with BQ_(aq)_ under quasi-steady condition (see Fig. 2a – 50 mV/s). Twice lower than in acetonitrile^21^, potential shifts are attributed to water as protic solvent, influencing both the kinetics (reactions involving proton transfers) and the thermodynamics (solubility via hydrogen bonding)^26^. PSS^­^_(aq)_ can also influence the redox products’ stability both as surfactant and dopant. CV shows that NaPSS_(aq)_ is not electrochemically active, implying that BQ_(aq)_ reduction is the counter-reaction for dendritic electropolymerization, without redox-active participation of PSS^−^.

With *V*_p_ ≥ 3.5 V, nucleation on both wires is observed at the minute scale (Fig. 2b at *V*_p_ = 3.5 V, |*V*^+^| = |*V*^−^| = 3.9 Δ*V*_min_). Lack of apparent bubbling evidences kinetic control, as such voltage bias promotes water electrolysis under steady conditions. The electro-generated object shows protuberances with a spatial periodicity that are uncorrelated with the signal’s periodicity (Fig. 2b). They are mechanically fragile and could hardly be pulled out of the electrolyte without damage. Scanning electron microscopy (SEM—Fig. 2c–e) shows their brittleness with sharp-edged cut, suggesting non-plastic properties for the polymer. Electrical tests show conductivity at the order of 0.1–1 S/cm (Supplementary Fig. 1), only 3-fold lower than optimized PEDOT:PSS dispersions^24^, and equals other highly doped polythiophenes’^27^. This evidences the presence of PSS^−^ into the ePEDOT matrix as dopant. SEM shows multi-scale morphological richness by apparent branchy structures at the 10–100 µm range, and sub-µm rough texturing (Fig. 2d, e). By varying *V*_p_ from 3.5 to 6.5 V at *f* = 80 Hz, dc = 50%, and *V*_off_ = 0 V, both higher-scale morphology and growth dynamics can be controlled (Fig. 2f–h): Time-lapse frame processing of microscope videos recorded for different growths shows the dendrite projected area increasing with time (2D-image of the 3D object) with strong influences of *V*_p_ (Fig. 2f). As waveforms are statistically symmetrical around ground (dc = 50%, *V*_off_ = 0 V), material grows evenly in size and morphology on both wires (Fig. 2h), except in the lower-voltage case, where it was experimentally observed that such lower limits are highly sensitive to the wire cutting. Growth is very slow from *V*_p_ = 3.5 V, and saturate at *V*_p_ = 5 V with a rate of 150 µm^2^/s. This indicates that the charge-transfer rate for either oxidation or reduction reaches an upper limit, yielding sensitively similar growths for *V*_p_ ≥ 5 V under these conditions. Discrete 1D-spatial decomposition of the 3D dendrite into an elementary cylinder (details in Supplementary Fig. 3) evaluate this upper limit to physically equal 3.68 × 10^3^ µm^3^/s, or 5.1 ng/s (assuming 1.4 g/cm^3^)^28–31^. Assuming an apex-growth over a specific area comprised between one and ten times the wire orthogonal cross-section, the surface rate is estimated around 0.1–1 mg/cm^2^/s, corresponding to 750-7500 nm/s for the equivalent case of an homogeneous thin-film coating. As such rate is at minimum 15 times higher than what is performed in potentiostatic electrodeposition of ePEDOT thin-films (we usually obtain only up to 50 nm/s for EDOT_(aq)_ in NaPSS_(aq)_ at 1.5 *V*_DC_ bias between two Au-electrodes), this suggests a higher turbulence in the electropolymerization mechanism under such waveforms leading to dendritic arborescences rather than films under such conditions. As growth rates differ with *V*_p_, dendrites also interconnect differently from 178 s at *V*_p_ = 5 V to 423 s at *V*_p_ = 3.5 V (Fig. 2f, h), systematically at the mid-distance of the inter-wire gap, to form a conductive bridge between both gold wires (Supplementary Fig. S4). Morphologies are also V_p_ dependent, as low-V_p_ growths have low fractality, and higher-*V*_p_ objects are more dendritic with apparent branching degree up to five. The branching factor and fractal dimension increase from 3.5 to 5 V and decrease afterward. These two trends show poor monotonicity, for which reproducibility tests showed high variability for these two numerically evaluated figure of merit, although completion time and projected area remain reproducible over trials, relatively to changes of 0.5 V in *V*_p_ (see Supplementary Fig. 2 for details).

For all *V*_p_, new ePEDOT nuclei were systematically seeded on the apex of freshly grown branches rather than from the periphery of the gold wires. This evidences of two behaviors: Field activation of the process (as in each case, dendrites appear to grow along the longitudinal wire-to-wire axis) and lower EDOT oxidation rate at the gold/electrolyte interface than the ePEDOT/electrolyte one (implying low resistance of gold/ePEDOT contacts and good conductivity of ePEDOT material). In this setup, field-effect sensitivity is justified by the influence of the anode-to-cathode distance that controls the ionic transport resistance 1/*G*, which in turn controls the voltage drop at both anode/electrolyte and cathode/electrolyte interfaces.

### Morphological influence with voltage offset

**Fig. 3:**
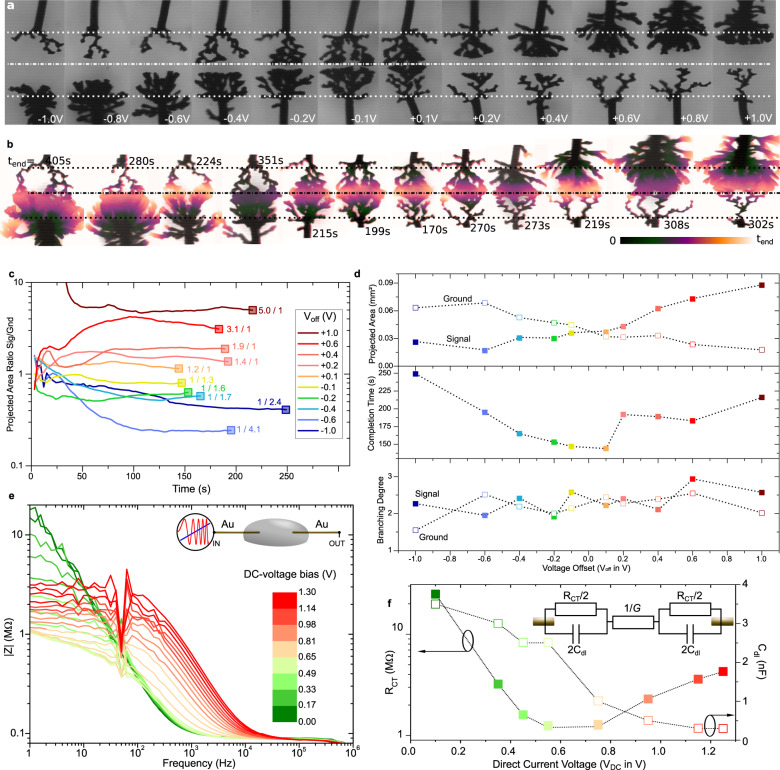
Asymmetry control by the voltage offset (*V*_off_). **a** Microscope pictures of ePEDOT dendritic formation after *t* = 150 s of voltage polarization under different *V*_off_ (*V*_p_ = 5 V, *f* = 80 Hz, dc = 50%). **b** Color-encoded temporal images of the same dendrites grown from 0 to *t*_end_ (color scale as inset as linear scale with time). **c** Ratio between the projected area of signal’s dendrite and ground’s over time, for dendrites grown at different *V*_off_. **d** Projected area, completion time, and branching degree over *V*_off_ for dendrites grown up until contact. **e** Voltage-ramped impedance spectroscopy between *V*_DC_ = 0 and 1.3 V of the two-gold-wire setup immersed in the same electro-active electrolyte (10 mM BQ_(aq)_, 10 mM EDOT_(aq)_, 1 mM NaPSS_(aq)_) that displays a bias-dependent high-pass filter property (the effect of an ePEDOT coating on either or both wires is provided as a Supplementary Material). **f** Extracted *R*_CT_ and *C*_dl_ parameters from the fitting of the spectrum on the idealized equivalent circuit depicted as an inset (*G* = 8.5 ± 0.2 µS). Scatters and curves’ color encodings with a voltage offset in **c** and **d** are identical. Scatters and curves’ color encodings with DC voltage in **e** and **f** are identical.

**Fig. 4:**
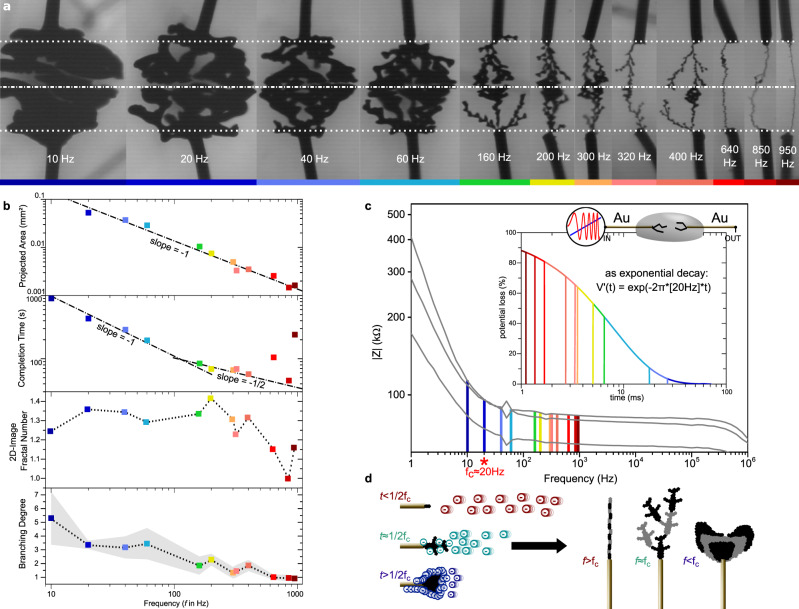
Fractal control by the frequency (*f*). **a** Microscope pictures of ePEDOT dendritic formations (signal wire: top, ground wire: bottom) at their completion time grown under various frequencies from 10 to 950 Hz (*V*_p_ = 5 V, *V*_off_ = 0 V, dc = 50%). **b** Projected area, completion time, fractal number of their image, and branching degree over *f* for dendrites grown up until contact. The gray-colored error interval on the branching degree is set to the mean value ± SD (six values along *L* for each *f*). **c** Impedance spectroscopy of the two-wire setup after three different dendritic formations (see details on the three evaluated dendrites as Supplementary Information), performed in 1 mM NaPSS_(aq)_ (without EDOT_(aq)_ nor BQ_(aq)_), that displays in the three cases a change in the frequency-dependent regime of the modulus at *f*_c_ around 20 Hz. Inset: Decay over time of the potential across the wire assuming an ideal resistor-capacitor discharge, and an exponential decay rate τ = 1/[2π*20 Hz]. **d** Schematics for the growth of three different dendrites with morphological dependencies associated to anionic screening during polarization responsible for the potential drop at the anode, for which the hindrance time dependency is associated to the frequency and the *f*_c_ of the electrochemical system. Scatters’ color encodings with frequency in **a**, **b**, and **c** are identical.

By introducing a voltage offset (*V*_off_) in the waveform, strong assymmetries are induced between both dendritic growth (Fig. 3). *V*_off_ influences the system by two distinctive ways. It generates anodic and cathodic voltage-asymmetry between both wires, which in turn leads to different electropolymerization rates for both wires. Also, different electric-fields amplitudes are imposed during both half-periods, with different field lines governing different growths orientation. From both effects, we distinguish both effects on the growth rate and its anisotropy to be influenced by *V*_off_ on the morphology. The sign of the offset controls the growth asymmetry: larger arborescences are systematically observed on the signal wire with positive offsets and on ground with negative offsets, with a monotonic trend in the gradual asymmetry control with |*V*_off_| (Fig. 3a, c). The timescale for the dendrites formation decreases with the increase of |*V*_off_| (Fig. 3b, c). Despite *V*_off_ induces electric fields with different magnitudes on the dendrites forming at both wires, both growths interconnects systematically at mid-gap. This shows that although the offset promotes the growth on one electrode over the other, the equatorial component for both growths (parallel with the electric field) remains comparable, with an equal growth velocity that is uncorrelated to the dendrite morphology. Both dendritic morphologies expand with distinctive modes: the thinnest dendrite’s appears to be the most linear and longitudinal with the equatorial direction, while the densest one shows always radial growth (Fig. 3b). This evidences that the thinnest dendrite (experiencing lower applied oxidation voltage) is more dominated by the electric field than the thickest one (experiencing higher applied oxidation voltage). Therefore, dendritic growths are independently governed by both the voltage amplitude at the electrode and their induced electric field. As depicted in Fig. 1c, wires behave both as anode and cathode during the experiment (as *V*_p_ > *V*_off_ in each case) and growths are occuring on both wires (as *V*_p_ − *V*_off_ > ∆*V*_min_ in each case). However, as the wire experiencing the highest oxidation voltage shall expose also to the highest field polarization, this shows that field orientation controls the growth only for low growth rates, as the thin dendrites appear straighter along the field orientation than thicker ones. The observed change in growth orientation evidences voltage non-linearity in the charge-transfer yielding anodic processes of electropolymerization on wires. Non-Ohmicity under quasi-steady conditions was evidenced by voltage-ramped impedance spectroscopy (Fig. 3f). As the direct-current voltage bias (*V*_DC_) is gradually increased over time in the experiment, we observed a sudden decrease in the charge-transfer resistance *R*_CT_ = *R*_ox_ + *R*_red_ when approaching *V*_DC_ = ∆*V*_min_, increasing with *V*_DC_ after reaching a minimum (Fig. 3f). Confirming the voltage activation of the growth, it also invalidates the linearity of the applied electric-field *E* = (*V*’_sig_ − *V*’_gnd_)/*d* (*d* < *L* as shortest distance between two dendrites), with *V*’_sig_ and *V*’_gnd_ being *R*_CT_(*V*_DC_) dependent, and therefore dependent on *V*_off_. Furthermore, we observed the growth continued further after interconnecting, by the appearance of radial dendrites on the thickest dendrite wire for higher *V*_off_ values (easily evidenced in Fig. 4b for time lapses at *V*_off_ = −0.4, +0.2, and +0.8 V recorded until *t*_end_ longer than their respective completion time). Such feature indicates good conduction for these asymmetric objects after merging: As none of the transmission lines were loaded with serial resistances on any sides (other than the 50 Ω protection of the generator and the <1 Ω of our coaxial setup), the electrical conduction is limited by the resistance of the fused dendrites, for which the higher value causes the voltage to drop all along both dendrites. Leaving the highest point of the applied potential to be strictly on the gold surface of the anodic wire, electropolymerization continues further after merging by the creation/expansion of new branches on gold.

### Morphological influence with frequency

The influence of the frequency using square waveforms on the growth of ePEDOT wires in a water/acetonitrile electrolyte has recently been shown^32^, and evidences an empirical law existing between the apparent diameter of the ePEDOT wires and frequency of the applied waveform. Here, we focused on a lower frequency range between 10 Hz and 1 kHz to monitor the change transition between dendrite-like to wire-like that occurs by increasing the frequency in water (Fig. 4). As the system starts to bubble for frequency below 10 Hz, this property confirms that the system is kinetically limited and water electrolysis is only enabled if the applied voltage remains still for a sufficiently long time.

Frequency variation in the voltage pattern shows a relationship between the projected area and the frequency that appears inversely proportional (as shows the −1 slope in double log scale in Fig. 4b), suggesting here that projected area is linear with the waveform’s period. As the projected area is directly proportional to the dendrite’s branches diameter by the elementary cylinders approximation (at least for the highest frequency cases where dendrites appear as linear cables), the quadratic low identified for lithographically patterned Au-electrodes in acetonitrile/water at higher frequency appears linear for our experimental conditions for dendritic growth. At higher frequency, dendrites’ seems also to interconnect quicker, by a linear relationship between completion time and frequency displaying a −1 slope in double log scale (Fig. 4b) at a frequency below 200 Hz. In case of lower branching dendrites above 200 Hz, dendrites grow slower, by a −1/2 slope on the double log plot in Fig. 4b. We observed a deviation from this trend for *f* > 200 Hz with substantially much thinner dendrites: dendrites interconnect much slower up to 950 Hz. Nuclei with micrometer-scale branches at a frequency above 1 kHz were observed but have not been attempted to merge at this wire distance, due to a too long completion time, not compatible with preserving the electrolytic drop from substantial evaporation. From the impedance spectroscopy of three different dendritic doublet morphologies (thin and vicinal, thick and vicinal, and thick and distant—details in Supplementary Fig. 5), all three pairs of dendrites have a comparable spectral behavior with a cut-off at around *f*_c_ = 20 Hz. Compared to the naked gold wire, all these dendritic systems take longer to charge their double layers (observed cut-off between 1 and 10 kHz for naked gold in Fig. 3e). With dendrites’ specific areas being much larger than the wire, the increase of the double-layer capacitance at the electrochemical interfaces is responsible for the lower frequency shift of the cut-off. Providing that this 20 Hz cut-off is similar for all three pairs of dendrites, we observe that the first 25 ms of the wire polarization appears to be a specific time window in the charge transient of dendrites of these sizes. Earlier than *t* = 1/2 *f*_c_, the ionic space-charge around the electrodes is not fully-formed as compact Helmholtz layers to screen the voltage polarization at the vicinity of the electrodes in the electrolyte (*V*’_sig_ near the signal and *V*’_gnd_ near the ground, as depicted in Fig. 1b). Assuming ideal resistor-capacitor charge/discharge for the PSS^−^_(aq)_ Helmholtz layer at the anode, a significant exponential decay of *V*’_sig_(*t*) in the millisecond timescale, which therefore conditions the oxidation rate of EDOT_(aq)_ in the sub-kHz range (Fig. 4c). As the correlation between spectral dependency growth morphology is not straightforward, the mechanistic interpretation of the dendrites’ growth morphology with voltage frequency remains only qualitative. However, in light of the spectral and image analysis, we can assess that the process is not limited by an anodic monomer gradient diffusion, as the most massive dendrites are obtained at the lowest frequency for a same polarization duration. As in this case, the temporal density of anodic polarization equals 0.5 (dc = 50%) for all frequency conditions, the densest dendrites would have been expected for the highest frequency in case of diffusion limitation. Also providing the ionomeric structure of the PSS^−^_(aq)_ polyanions, one can assume that its time-dependent accumulation on the ePEDOT surface screens the dendrites both electrostatically but also sterically. Such physical hindrances for the EDOT_(aq)_ oxidation can potentially justify the observed gradual transition of morphology from isotropic and diffused growth to oriented and linear ones (Fig. 4d).

### Engulfing asymmetry with the duty cycle

**Fig. 5:**
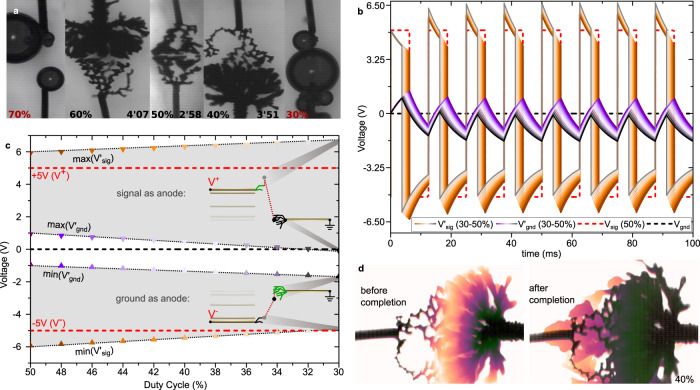
Dendritic engulfing and asymmetry with the duty-cycle (dc). **a** Microscope pictures of ePEDOT dendritic formations (signal wire: top, ground wire: bottom) at completion time after growth under different duty cycles (*V*_p_ = 5 V, *V*_off_ = 0 V, *f* = 80 Hz—the two extreme cases dc = 30 and 70% where taken at *t* = 1 min). **b** Simulation of the *V*’_sig_(*t*) and *V*’_gnd_(*t*) dynamics under a pulse waveform (*V*_p_ = 5 V, *V*_off_ = 0 V, *f* = 80 Hz) with various duty cycles between 50 and 30% (assuming the equivalent circuit of Fig. 4f inset with rigid parameters as *R*_CT_ = 2 MΩ, 1/*G* = 80 kΩ and *C*_dl_ = 10 µF). **c** Dependency of *V*’_sig_ and *V*’_gnd_ voltages at the change of the signal polarity (spike) upon variations of the waveform’s duty cycle between 50 and 30% from simulations displayed in Fig. 6c (the two diagrams show the different voltage drops between the dendrite at both changes of the waveform’s polarity for dc = 30%). **d** Color-encoded images of a dendritic growth at dc = 40% showing two specific dynamics before and after completion. Scatters and curves’ color encodings with a duty cycle in **b** and **c** are identical.

The duty-cycle parameter singularly differentiates square waveforms (dc = 50%) from generic pulses. By modulating dc by ±10% around 50%, comparable asymmetric trends are observed as previously with changing *V*_off_ (Fig. 5a): At 60%, the signal’s dendrite is denser than the ground’s and vice versa at 40%, as the growth is enhanced on the wire experiencing the longest anodic duration. The relationship between dc and *V*_off_ is quantified in the Fourier decomposition of the pulse waveform (Eqs. 1 and 2)^33^,

1 $${{{V}}}_{{{{{{\rm{sig}}}}}}}\left(t\right)-{{{V}}}_{{{{{{\rm{gnd}}}}}}}={{{V}}}_{{{{{{\rm{off}}}}}}}+(2{{{{{\rm{dc}}}}}}-1){{{V}}}_{{{{{{\rm{p}}}}}}}+\mathop{\sum }\limits_{n=1}^{{{\infty }}}\frac{4{{{V}}}_{{{{{{\rm{p}}}}}}}}{n{{\pi }}}{{\sin }}\left({{\pi }}n{{{{{\rm{dc}}}}}}\right)\cdot {{\cos }}\left(2{{\pi }}{nf}\cdot t\right)$$

2 $${{{{{{\rm{V}}}}}}}_{{{{{{\rm{sig}}}}}}}\left(t\right)-{{{{{{\rm{V}}}}}}}_{{{{{{\rm{gnd}}}}}}}={{{{{\rm{dc}}}}}}{{{{{{\rm{V}}}}}}}^{+}+(1-{{{{{\rm{dc}}}}}}){{{{{{\rm{V}}}}}}}^{-}+\mathop{\sum }\limits_{n=1}^{{{\infty }}}\frac{2({{{{{{\rm{V}}}}}}}^{+}-{{{{{{\rm{V}}}}}}}^{-})}{n{{\pi }}}{{\sin }}\left({{\pi }}n{{{{{\rm{dc}}}}}}\right)\cdot {{\cos }}\left(2{{\pi }}{nf}\cdot t\right)$$

Where introducing an asymmetry of the temporal polarization densities by dc allows inserting a time-invariant term *V*_DC_ in the waveform without breaking the applied-voltage asymmetry of the *V*^+^ and *V*^−^ around zero, such that (Eq. 3):

3 $${{{{{{\rm{V}}}}}}}_{{{{{{\rm{DC}}}}}}}={{{{{{\rm{V}}}}}}}_{{{{{{\rm{off}}}}}}}+(2{{{{{\rm{dc}}}}}}-1){{{{{{\rm{V}}}}}}}_{{{{{{\rm{p}}}}}}}={{{{{\rm{dc}}}}}}{{{{{{\rm{V}}}}}}}^{+}+\left(1-{{{{{\rm{dc}}}}}}\right){{{{{{\rm{V}}}}}}}^{-}$$

From the Fourier expression of the pulse wave, *V*_DC_ equals a sum of *V*_off_ and a fraction of *V*_p_, such that a dc = 60% generates *V*_DC_ = 1 V, 2 V at 70%, −1 V at 40%, and −2 V at 30% (for *V*_p_ = 5 V, *V*_off_ = 0 V). The dependency of the projected area ratio signal/ground *ρ*(dc,*V*_off_) with the duty cycle illustrates this relationship in our experiments: at completion, time *ρ*(40%, 0 V) = 0.18 and *ρ*(60%,0 V) = 3.11, while previously we observed *ρ*(50%,−0.8 V) = 0.24 and *ρ*(50%,+0.8 V) = 3.1 (Fig. 3c), suggesting that the dc component in the waveforms rules the dendrites’ asymmetry in both *V*_off_ and dc variation series. This evidences that the electropolymerization occurs at double-layer charging and not in the electrolytic conduction regime. This further confirms the frequency increase results in a lower projected area. In terms of circuit-element analysis, the *V*_DC_ control by the dc originates from the temporal asymmetry of the charge/discharge processes at both electrode, resulting in the partial charging of one specific ion type on both electrodes favored by the longer-lasting polarization. Specific to dc (previously *V*_p_, *V*_off_, and *f* did not promote such effect), charge builds-up at the double-layer capacitors of both dendrites, inducing a dc-dependent serial potential pinning. From the electric simulation of the first-level approximated equivalent circuit (Fig. 3f, inset), one can observe that such a charge build-up induces a transient dc-dependent over-potential at both electrode interfaces that can be higher in absolute value than the applied voltage (Fig. 5b). At the beginning of the experiment (*t* = 0 in Fig. 5b), both double layers are unformed around the wires, so the initial state of their corresponding capacitor are fully discharged and induce exponential decays with *V*_sig_ and *V*_gnd_, as starting values of their surface potentials. At the second polarization change (*t* = *T*^+^ = dc*12.5 ms in Fig. 5b), the electrostatic charge/discharge of the double-layer capacitors initiate from different values than *V*_sig_ and *V*_gnd_ because of the partial charge by past polarizations. So in Fig. 5c, we observe that |*V*’_sig_| > max(|*V*^+^|;|*V*^−^|) and *V*_gnd_ ≠ 0 at the polarization inversions in the applied voltage wave, and therefore the generated transient electric-fields across the electrolyte are much higher in magnitude than the external *V*_sig_/*L* applied on the gold wires (insets in Fig. 5c graphs). While this phenomenon is commonly induced in each of the previously studied cases, the asymmetry on the duty cycle allows stretching the time windows for one preferential ionic charge/discharge over the other at each electrode, which in turn modifies the initial potential pinning of the initial state of the charge/discharge at each potential inversion. One can notice in Fig. 5b that although the duty cycle does not modify the decay rate by decreasing dc from 50 to 30%, it changes the initial values from +5.9 V/−5.9 V for *V*’_sig_ and +0.9 V/0.9 V for *V*’_gnd_ at dc = 50% to +6.7 V/−4.9 V for *V*’_sig_ and −1.7 V/−0.1 V for *V*’_gnd_ at dc = 30% (Fig. 5b, c). As in Eq. 3, this effect is linear with the duty cycle (Fig. 5c), and can even reach states where the surface potential at the ground electrode keep the same polarity vs. gnd over time (see dc = 30% in Fig. 5c, where max(*V*’_gnd_) crosses the *V* = 0 line): Although one gold wire is grounded at *V*_gnd_ = 0 V, its surface potential *V*’_gnd_ at its dendrite/electrolyte interface under strong *T*^+^/*T*^−^ asymmetry creates a permanent formation of exclusively one kind of ion. In Fig. 5c, the simulation shows also that the voltage drop *V*’_sig_ − *V*’_gnd_ across the electrolyte at the polarization change is strongly asymmetric by varying dc, with the initial values (max[*V*’_sig_] − min[*V*’_gnd_])_Vsig=*V*+_/(min[*V*’_sig_] − max[*V*’_gnd_])_Vsig=V−_ = +6.9 V/−6.9 V at dc = 50% and +8.3 V/−4.8 V at dc = 30%. This implies that under an asymmetric T^+^/T^−^ a regime with the dc, the thinnest dendrite grows under a larger field than the thickest one (insets of Fig. 5c), confirming that the thinnest dendrites seem more unidirectional along the inter-wire axis than the bulkiest radial dendrite in a same experiment (Fig. 5a). Finally, we experimentally observed that the dendrites grown under such an asymmetric T^+^/T^−^ regime have a particularly peculiar behavior after the completion time, as they continue to propagate along the wires and densify the connection in polymer material (Fig. 5d). In the previous experiments with dc = 50% for the study of the influence of *V*_p_, *V*_off_, and *f*, all dendrites converged to the mid-gap with difficulty to interconnect (as in Fig. 3b where growths after completion creates new branches on the gold edge). Instead, we observe that modulating the duty cycle allows forming more robust interconnections, with the two wires welded together by the thickest ePEDOT dendrite engulfing the thinnest one after completion.

### Program sensing-array via artificial dendritic morphogenesis

**Fig. 6:**
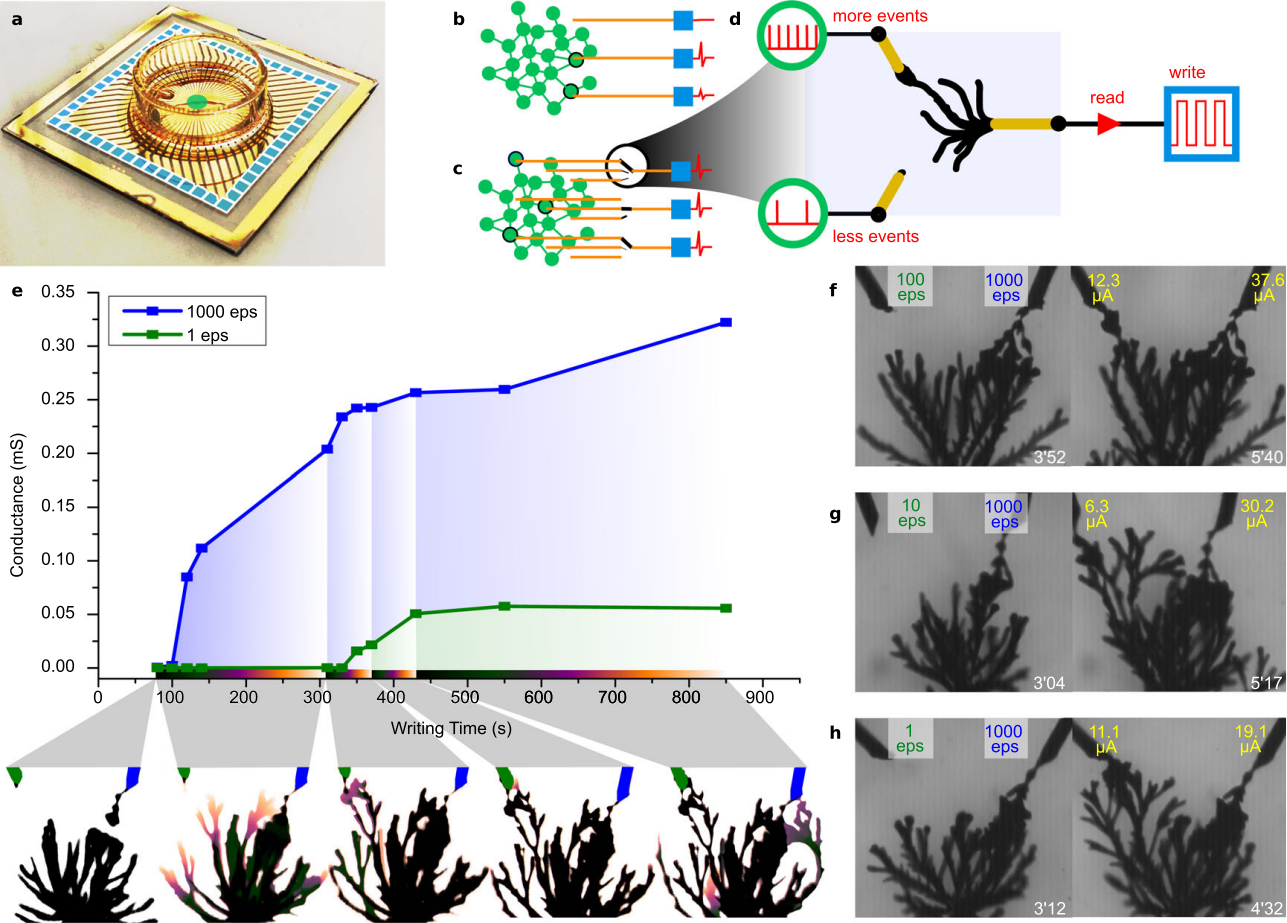
Hardware-based sensing-array dimensionality reduction via event-based dendritic interconnectivity. **a** Multi-Electrode Array (MEA) as an example for high-dimensional electro-sensitive hardware with no computing layer between the neural culture (input in green) and the restricted number of electrical contacts (output in blue). **b** Illustration for the poor readout of an unprogrammable array to efficiently sense active cells and collect relevant information. **c** Concept of using electrical event-based dendritic interconnectivity to program an array to self-connect an output to the most electro-active inputs. **d** Using electropolymerization as an electrochemical-PROM, the morphology created upon the polarization of the output to an appropriate waveform (writing), shall promote a specific morphology that favors earlier and higher conduction (read) interconnectivity towards the sensing input that has experienced most unipolar activity. **e** Temporal evolution of the conductance of a three-wire junction (2INx1OUT), where both inputs are stimulated at a different number of events per second (eps, one event being 200 mV vs. GND for 0.5 ms) and the output wire stimulated at *V*_p_ = 2.9 V, *V*_off_ = 1.1 V, *f* = 80 Hz, dc = 40%. At the bottom is displayed the color-encoded images of the growths displaying the different phases of completion responsible for the fastest-and-highest interconnectivity of the output to the input that experiences the highest number of event per second. **f**–**h** Three-wire junction (2INx1OUT) growths that compare the interconnectivity competition between one sensing input experiencing 1000 eps (200 mV vs. GND, 0.5 ms) and one sensing input experiencing 100 eps (200 mV vs. GND, 0.5 ms **f**), 10 eps (200 mV vs. GND, 0.5 ms **g**) and 1 eps (200 mV vs. GND, 0.5 ms **h**) with output continuously polarized at *V*_p_ = 2.9 V, *V*_off_ = 1.1 V, *f* = 80 Hz, dc = 40%. Scatters, curves, and labels’ color encodings within **e**, **f**, **g** and **h** are identical, so green depicts the electrode experiencing the lowest rate of activity, while blue depicts the electrode experiencing the highest rate of activity in eps.

Each waveform parameter rules specific properties for the growths and morphologies. Reciprocally, unique dendritic interconnections can be generated with specific dynamics engraving the spike voltage history experienced between two terminals. Electropolymerization’s irreversibility suggests both high stability for the interconnects, and immutability to permanently engrave information-pattern history. Standalone as electrochemically-programmable read-only memory, such a mechanism could relevantly be exploited on high-dimensional-sensing devices where information classification is limited by both the sensed pattern complexity (input layer dimensionality) and application incompatibilities to embed conventional computational resources on-board (integration issues, substrate requirements, biocompatibility). Typically, neurosensing micro-electrode arrays (MEA) are of such electro-sensitive hardware requiring transparent and biocompatible substrate, and where large number of inputs are singly-connected to outputs without information transformation (Fig. 6a): aiming sensing highly dense inputs of 10^3^ cells/mm^2^ ^34^, practical ability to sense and process in parallel enough of them severely reduces information dimension to classify neural activity (Fig. 6b). Based on spike-event activation, such electropolymerization process would promote Hebbian plasticity inside gold-electrode tracks as a hidden layer(s): growth of electrical interconnects occurs only with a dynamical correlation of a well-defined output waveform with sensed input perturbations that are on the edge of activating specific growth. This yields to selective interconnection, specifically on the sensed voltage patterns with interconnection morphologies and completion time (Fig. 6c–d). To demonstrate the impact on interconnections’ conductance, an elementary three gold-wire setup constituting a two-dimensional input and a single output have been evaluated (Fig. 6e–h). Output is programmed with a waveform (*V*_p_ = 2.9 V, *V*_off_ = 1.1 V, *f* = 80 Hz, dc = 40%) designed to be perturbed by inputs with low voltage spikes (*V*^+^ = 0.2 V, *t*^+^ = 500 μs, *V*^−^ = 0 V), firing at different spiking rates and resting at zero volt. As such, interconnection mechanism is not solely based on the capability of sensed input spikes to generate dendrites, but how they perturb a dendritic growth with an output writing waveform, so different morphology conditions the interconnection based on the input rates. Reading can be performed independently from writing thanks to electropolymerizations’ voltage non-linearity with *V*_DC_ < Δ*V* required for electropolymerization (we noticed that interrupting the writing sequence for reading dendrites’ conductance state does not alter the growth). Figure 6e shows that under such polarization, the output’s large dendrite expands continuously towards both inputs, but connects the input that experiences most of the electrical activity (Fig. 6e, *t* = 255 s) prior to connecting the other one (Fig. 6e, *t* = 635 s). After output completes connecting both inputs, the conductance of the output with the low rate input remains always lower than with the higher-rate input (Fig. 6e). Interconnectivity is gradual as further maturation can strengthen the output conductance with the input that experiences higher rate. This was verified for different rates between 1000 eps, comparatively with 100, 10, 1 eps (Fig. 6f, g), demonstrating systematically faster and higher interconnectivity of the output with the highest input rate. As spikes are smaller than Δ*V*_min_, applicability is not limited to spiking activity enabling EDOT’s oxidation with BQ (smaller spikes would require more stable setup and finer tuning of the output waveform), although input spikes used here are only about twice higher than neuron’s action potential (110 mV in amplitude and duration about 1 ms)^35,36^. A high level of stochasticity for the morphologies remains due to bottom-up variability for dendrite fabrication, with no monotonic dependency between conductance and completion time performed on different experiments with different event rates (Fig. 6f–h). This shows that although event-based programmation can be set to classify inputs locally, their larger sensitivity to other parameters (temperature, concentration) conjunct to bottom-up randomness enriches both sensing dimensionality and computing reservoir’s complexity to classify complex environmental patterns with an electrochemical wetware. Dendritic morphogenesis exploits here only one aspect of neural plasticity: Biomimetically, no reverse “neurodegeneration” serves resetting the neural topology as a long-term memory mechanism (though threatening dendrite’s structural integrity to mimic cell death programming can be designed by electrolyte formulation, if mimicking it would serve pattern classification). However if individually regarded as PEDOT: PSS-based devices^3,37^, embedding synaptic plasticity with dendritic morphogenesis can enable reprograming the hardware for sparse machine learning with spiking ANN, for a new sensing paradigm where sensors behave as true sensory neural systems.

## Discussion

Dendritic electropolymerization of EDOT monomer in water has been evidenced here to be an electrochemical process that mimics neural dendrite arbor morphogenesis with a large versatility to tune electrical interconnection properties. Voltage offset, amplitude, frequency, and duty cycles of applied voltage spikes promote specifically the growth of a conducting-polymer bridges. Dendrites’ branching number, orientation, asymmetry, engulfing, surface, and length of the dendritic segments are ruled by the spike parametrizing, and the different electrochemical processes undergoing at different time domains on the different interfaces. Morphologies of such conducting bridges yield the impedance property by specific frequency-dependent resistance and capacitance that engrave the experienced voltage pattern in a unique-topology dendritic-interconnect. Narrowed to voltage parametrizing that are compatible with 5 V logic and frequencies far below any microcontroller clocks currently used in digital electronics, this study shows the relevance of such an electrochemical process for its integration in the largest electronic application field. Specifically for sensing, artificial morphogenesis showed its relevance to efficiently hard-wire input-output of electro-sensitive electrode based on voltage-event occurrence to specifically engrave spatiotemporal activity at the level of hundreds of millivolts. Such process can relevantly be used for any high-dimensional electro-sensitive sensing array, where information classification’s complexity can drastically be lowered in operando at the wetware level by gradually decreasing the device networking sparsity using voltage-event programming. Although this study points out structure-property relationships existing between polymer dendritic morphologies and the dynamical voltage history experienced across them, a high level of stochasticity in their arborescence remains. As the dynamical voltage dependency can serve understanding how translating learning rules to program their interconnectivity, the lower-scale material disorder engraved in the interconnects demonstrates high-potential to embed truly-aleatory function thanks to such object’ unclonability. Applied as analog-front-end to encode sensed signals into hardware morphogenesis that cannot be reverse-engineered, artificial dendritic morphogenesis shall greatly decrease the vulnerability of computing systems that requires both higher level of security and computing power to process personal data in new biometric sensing technologies^38^.

## Methods

### Materials and instrumentation

Dendrites were grown by bipolar alternating current electropolymerization technique in an aqueous electrolyte containing 1 mM of poly(sodium-4-styrene sulfonate) (NaPSS), 10 mM of 3,4-ethylenedioxythiophene (EDOT) and 10 mM of 1,4-benzoquinone (BQ). All chemicals were purchased from Sigma Aldrich and used without any prior purification. Two 25 μm-diameter gold wires (purchased from GoodFellow, Cambridge, UK) were employed as working and grounded electrodes, immersed into a 20 µl drop of electrolyte placed onto a Parylene C-covered glass substrate. To keep this study as systematic as possible and not observing morphological control of the dendrite due to the environment, cleaned gold wires have been lifted up to the same height above the same electrolyte-supporting substrate. The wire ends have been kept still and distant by *L* = 240 μm. The gold wires used in each experiment were not encapsulated with any passivating coatings, and the wires’ active surface/distance *S*/*L* only kept controlled by the micromanipulated setup stability and the controlled volume of the electrolyte with a micropipette on a clean surface. Though variabilities in equivalent capacitance C_dl_ and conductance 1/*G* could result from varying *S*/*L* (see Supplementary Fig. 6), the compensation of the one on the other does not change the cut-off position on the impedance at 2πC_dl_/*G*, not affecting the dynamical voltage dependency of the growth nor the dendrite morphology. Square-wave signals were generated from a 50MS/s Dual-Channel Arbitrary Waveform Generator (Tabor Electronics), with a consistent study of electrical parameters’ impact: peak amplitude voltage (*V*_p_), frequency (*f*), duty cycle (dc) and offset voltage (*V*_off_). Each dendrite growth was carried out with unused gold wires and daily prepared solutions.

### Image processing

Growths were recorded with a VGA CCD color Camera (HITACHI Kokusai Electric Inc). Video preprocessing involves greyscale-256 at 1 fps data conversion (with the VirtualDub open-access software), prior single-frame image processing (with the ImageJ open-access software). Each frame during the dendritic growths was linearly transformed with the StackReg and TurboReg ImageJ addons^39^, to compensate the time-dependent optical aberration caused by the electrolyte drops changing its curvature overtime due to partial evaporation. Furthermore, frames were cropped around the dendrites and binarized black/white with an appropriate user-defined contrast setting for the different videos generated for each growth. Time-lapses were color encoded with the corresponding ImageJ built-in tool to encode the dynamics of the growth on a scale from a dark purple (for the beginning of the growth), to light yellow (for the end of the growth).

### Feature extraction

The image parameters were calculated in Python, involving binarization, pixel counting, fractal dimension determination, branch calculation, and volume determination for the different electrical conditions. Dendrite density was obtained based on the pixel counting from the binarized images without resolution reduction of the original video frames sampled overtime (details in Supplementary Fig. 3). Error bars on graphs are based on the statistics of four different regions along the dendrite from the electrode to center. The extrapolated three-dimensional volume was calculated by assuming projected branches to have cylindrical structures, with the cylindrical diameter being equal to the branch width and the cylindrical height being equal to the branch length. The fractal dimension was obtained by box-counting algorithm by counting number of boxes and it quantifies the rate at which an object’s geometrical features develop at increasing resolution. For asymmetry determination, the image analysis process was individually performed on each dendrite at both ground and signal electrodes to compare their corresponding density, branches and fractal dimension. Inter-dendrite spacing was determined over time considering their extreme points from both sides, and completion time was defined as the spacing becomes zero. The overall discussion on the two-dimensional image analysis for the three-dimensional dendritic growths acknowledges that the method does not extrapolate three-dimensional physical properties, but serves as a systematic technique to reliably quantify two-dimensional features of three-dimensional objects.

Cyclic voltammetry was performed on a three electrodes setup, with the 25 μm-diameter gold wires as working and counter electrodes, and a macro Ag/AgCl reference electrode. Measurements were performed distinctively for the solutions containing: 1 mM NaPSS_(aq)_, 10 mM BQ_(aq)_ and 1 mM NaPSS_(aq)_, 10 mM EDOT_(aq)_, and 1 mM NaPSS_(aq)_. For each experiment, a new pair of gold wires was used.

Electrochemical impedance spectroscopy (EIS) was performed on two gold wire systems with a Solartron Analytical (Ametek) impedance analyzer from 1 MHz to 1 Hz. Different gold electrode systems where characterized with and without dendrite functionalizations.

Systems without dendrite were characterized by voltage-ramped impedance spectroscopy (*V*_DC_ from 0 to 1.3 V at 1 mV/s, *V*_a_ =20 mV_rms_) in the same electrochemical conditions as for the dendritic growths: 10 mM BQ_(aq)_,10 mM EDOT_(aq)_ and 1 mM NaPSS_(aq)_. During the characterization, we observed by microscopy in operando the anodic growth by the appearance of a black coating on the whole surface of the polarized wire. The ePEDOT-coated gold electrode was used to study the electrochemical influence of the ePEDOT material on the impedance of pair of gold wire: the coated wires were re-used as anode, cathode, and/or both on subsequent EIS characterization (details in Supplementary Fig. S4). The thickness of the coatings was uncontrolled and not evaluated.

Systems with dendrite functionalizations were studied as two-electrode systems by impedance spectroscopy with a constant bias (*V*_DC_ = 100 mV, *V*_a_ =20 mV_rms_) in an electrochemically inactive aqueous electrolyte containing only 1 mM NaPSS_(aq)_, to not promote further electropolymerization of ePEDOT on the dendritic objects in operando characterization. In all three cases, dendrites were grown before completion and a priori, in the same electrochemical conditions as previously described. Three different morphologies were characterized: one pair of dendrites that are wire-like, one where dendrites are branchy and vicinal, and one where dendrites are branchy and distant (details in Supplementary Fig. S5).

Circuit impedance modeling was performed using an open-source EIS Spectrum Analyzer software^40^. The fitting was realized on the raw data spectra without digital-filter preprocessing. The RC parameter fitting was manually adjusted at the visual appreciation of the simultaneous comparison of the Nyquist plots, Bode’s modulus, and Bode’s phase plots.

Electrical circuit simulation was performed using the Quite Universal Circuit Simulator (QUCS) open-source software (http://qucs.sourceforge.net/), based on a (*R*|*C*) + *R* + (*R*|*C*) model, with voltage-independent parameters (ideal ohmic resistors and capacitors).

## Supplementary information

Supplementary Information

## Data Availability

The data generated in this study^41^, that include raw videos of dendritic growths, raw cyclic voltammograms, raw direct-current electrical characteristics, raw impedance spectra, their fitting parameters, 2D-image processed computed parameters (projected area, completion time, 2D fractal number, and branching degree), and circuit compact modeling data, have been deposited in the Figshare Repository database [10.6084/m9.figshare.16814710].

## Code availability

The Python codes for feature extraction are available from the authors upon reasonable request.
