# Supplementary material for: Analog programing of conducting-polymer dendritic interconnections and control of their morphology
Source: Nat Commun. 2021 Nov 25;12:6898. doi: 10.1038/s41467-021-27274-9 (PMC8617192; doi:10.1038/s41467-021-27274-9)
Supplement: Supplementary file 1 — Supplementary Information [file 41467_2021_27274_MOESM1_ESM.pdf]

Supplementary Information:

Analog Programing of Conducting-Polymer Dendritic Interconnections and Control of their Morphology

Kamila Janzakova[1], Ankush Kumar[1], Mahdi Ghazal[1], Anna Susloparova[1], Yannick Coffinier[1], Fabien Alibart[1,2] & Sébastien Pecqueur[1,*]

[1]Univ. Lille, CNRS, Centrale Lille, Univ. Polytechnique Hauts-de-France, UMR 8520 - IEMN, F-59000 Lille, France.

[2]Laboratoire Nanotechnologies &Nanosystèmes (LN2), CNRS, Université de Sherbrooke, J1X0A5, Sherbrooke, Canada.

Email: sebastien.pecqueur@*iemn*.fr

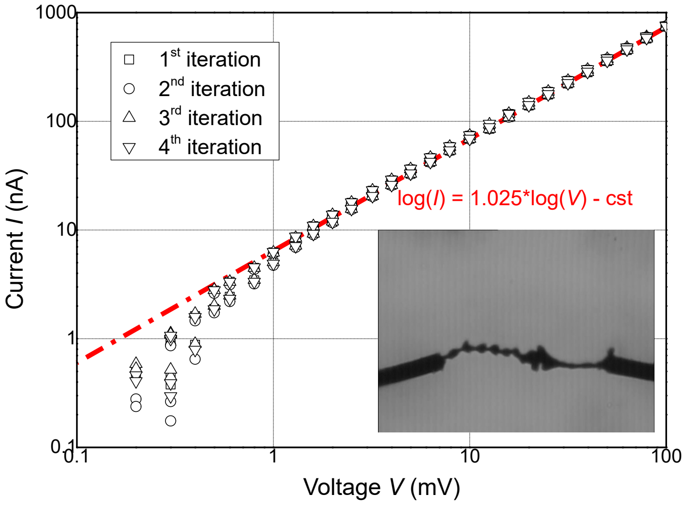

Supplementary Fig. 1 | Electrical Characterization of an ePEDOT dendritic formation. Four repeted iterations of output current characterization of the dendrite (displayed as an inset, grown with V_p = 3.5 V, V_{off} = 0 V, *f* = 80 Hz, dc = 50%) upon DC voltage polarization at t he wires, showing a Ohmic behaviour for the microstructure (voltage exponent close to one). The constant (cst) value of 5.1 (*I* in ampere and *V* in volt) indicates a conductance for the microstructure of 7.4 µS (resistance of 135 kΩ). Considering the length of the dendritic wire of 240 µm and an apparent section between 2 to 10 µm, the conductivity of the material is evaluated to be between 0.21 and 5.64 S/cm (assuming it to be uniform).

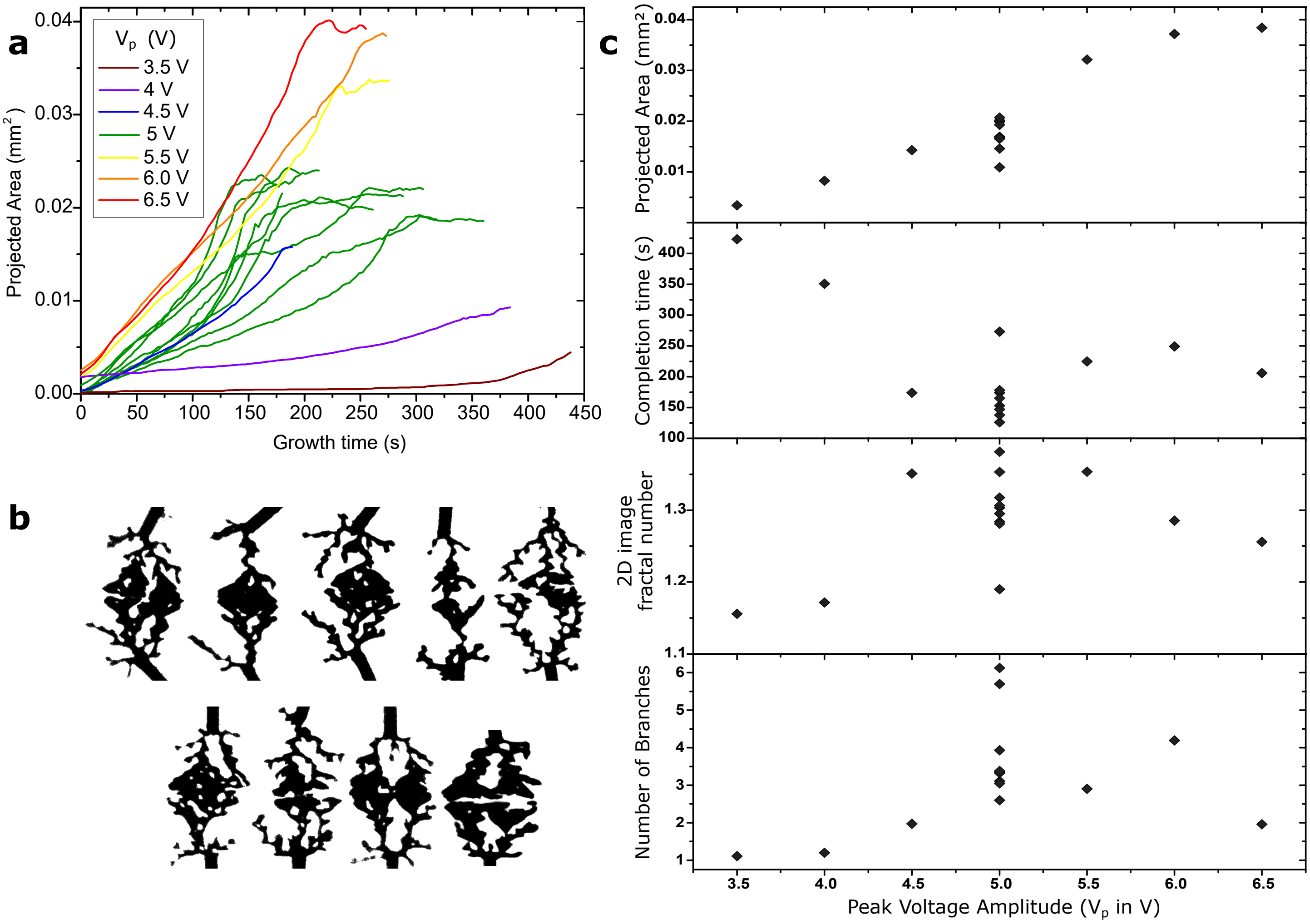

Supplementary Fig. 2 | Morphological Variability at fixed waveform parameters. a, Dendrites' projected area over time for distinct dendrites grown at different V_p variation (V_{off} = 0 V, *f* = 80 Hz, dc = 50%) and nine individual trials repeated at V_p = 5 V. **b,** Contrasted microscope images of nine dendritic morphologies grown under the same votage waveform (V_p = 5 V, V_{off} = 0 V, *f* = 80 Hz, dc = 50%). **c,** Relationship between V_p and projected area, completion time, their image's fractal number and the apparent number of branches for dendrites grown up to completion time (V_{off} = 0 V, *f* = 80 Hz, dc = 50%).

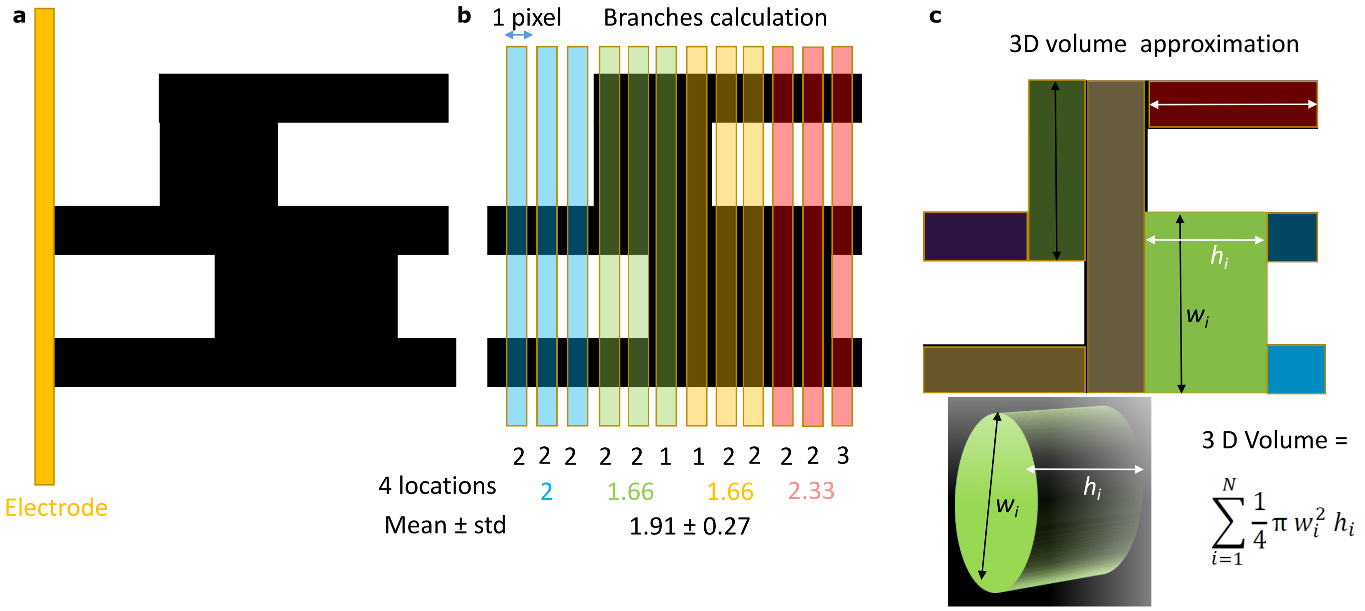

Supplementary Fig. 3 | Binarized Image Pixel Counting. a,b, Branching number was determined based on total intersections of dendrite structures with elementary vertical lines as pixels. **c,** Extrapolated three dimensional volume was calculated assuming the dendrite branches to have cylindrical elementary structures, with the cylinder diameter to be equal to the branch width and the cylinder height to be equal to the branch length.

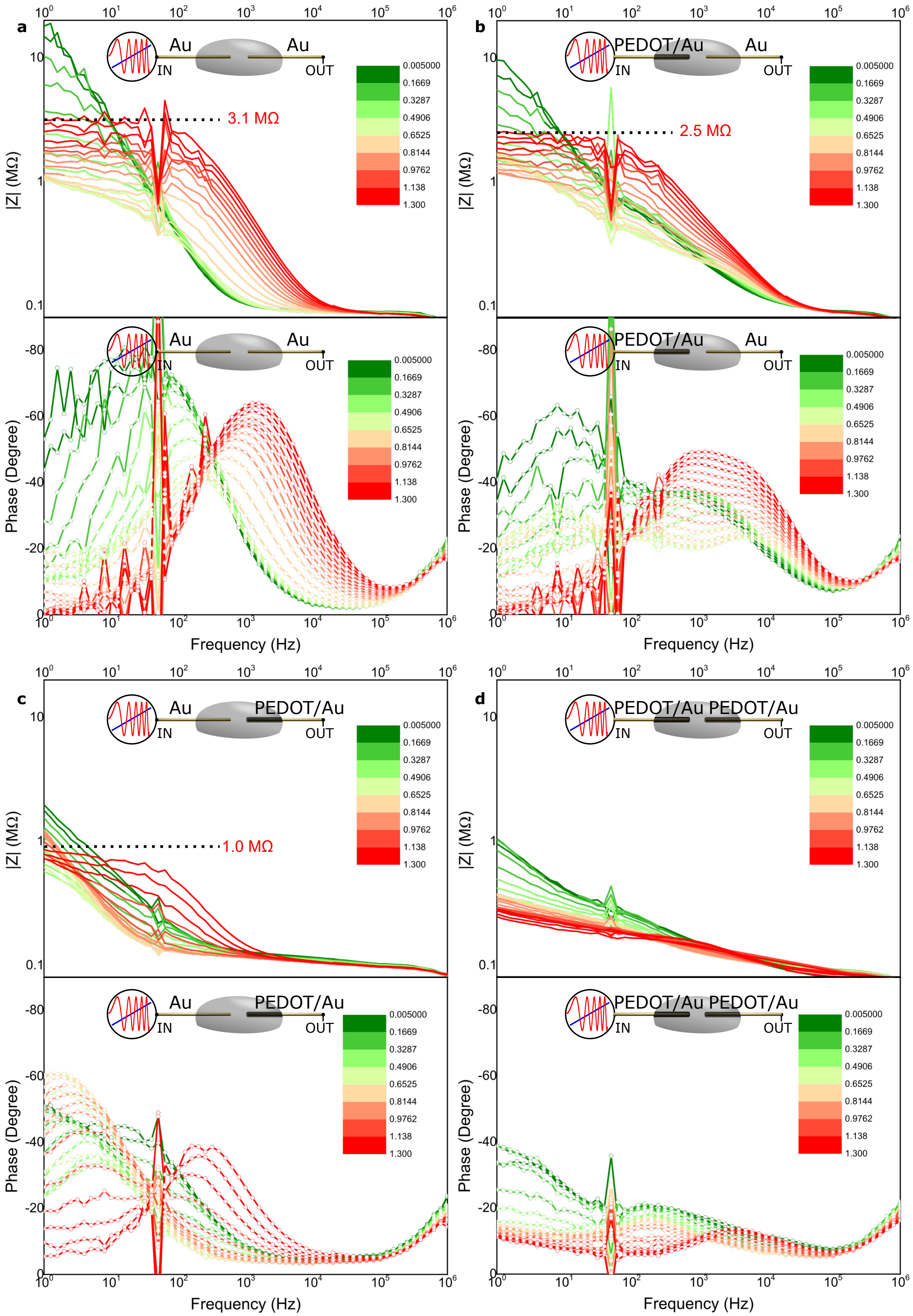

Supplementary Fig. 4 | Voltage-Ramp Impedance Spectroscopy on Au Wires. Bode impedance modulus and phase diagrams of naked gold wires (**a**), with a PEDOT coating exclusively on the input-wire (**b**), or on the output one (**c**), and on both wires (**d**). The spectra were generated in a comparable setup as the studies performed with square waveforms (1 mM $NaPSS_{(aq)}$, 10 mM $BQ_{(aq)}$, 10 mM $EDOT_{(aq)}$, 240 µm) with a DC voltage ramp (V_{DC}) from 0 to 1.3 V of 1 mV/s (color scale of V_{DC} in volt).

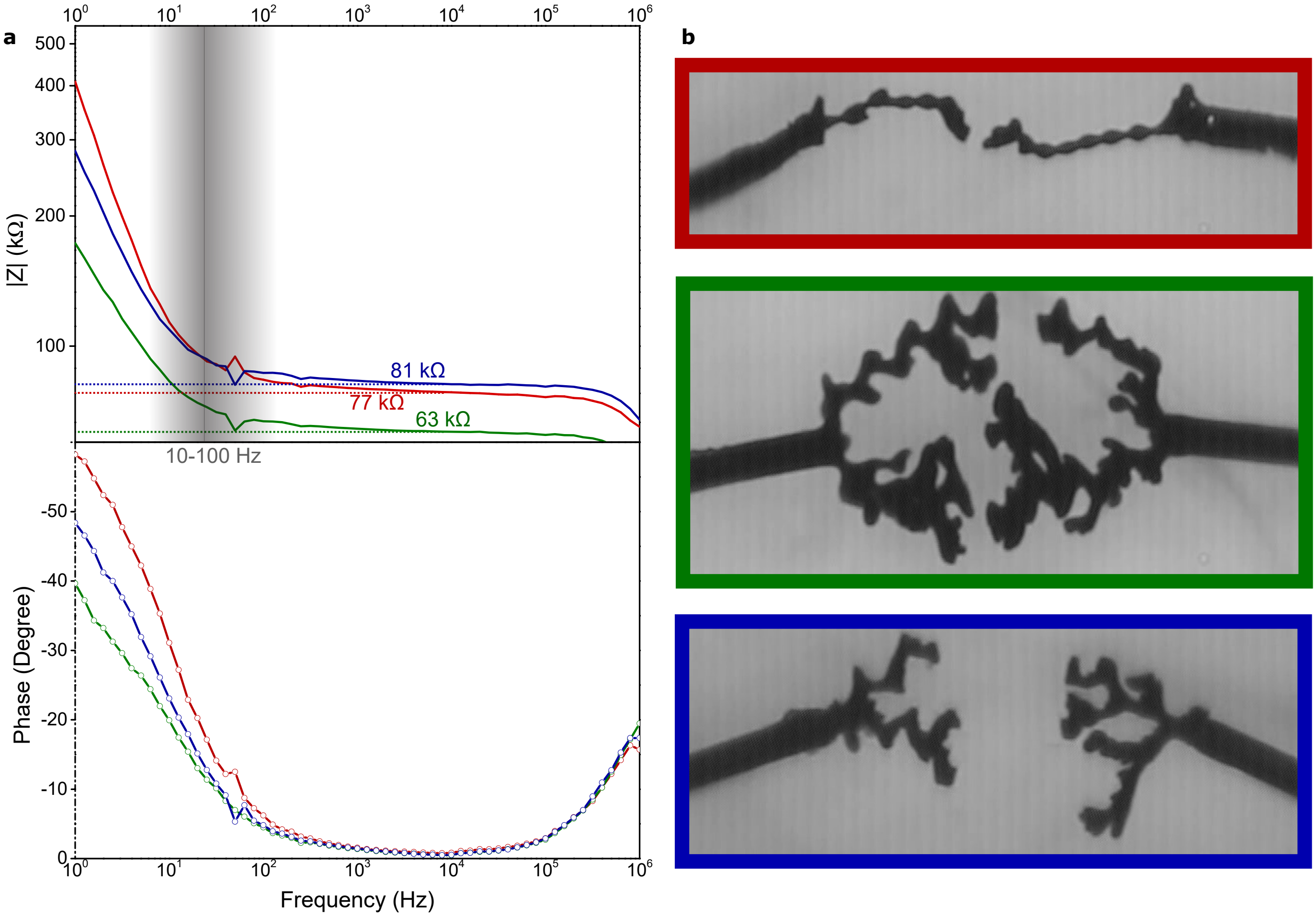

Supplementary Fig. 5 | Impedance Spectroscopy on Dendritic Structures. a, Bode impedance modulus and phase diagrams of dendritic structures grown on gold wires (distance: 240 µm): a narrower-gap & wire-like structures (red), narrower-gap & fractal dendrites (green) and wider-gap & fractal dendrites (blue). The grey shaddow indicates one decade frequency range used for the dendritic growth. **b**, Microscope picture showing the morphology of the three dendrites characterized by impedance spectroscopy (the .colour frames in **b** matches data's in **a**).

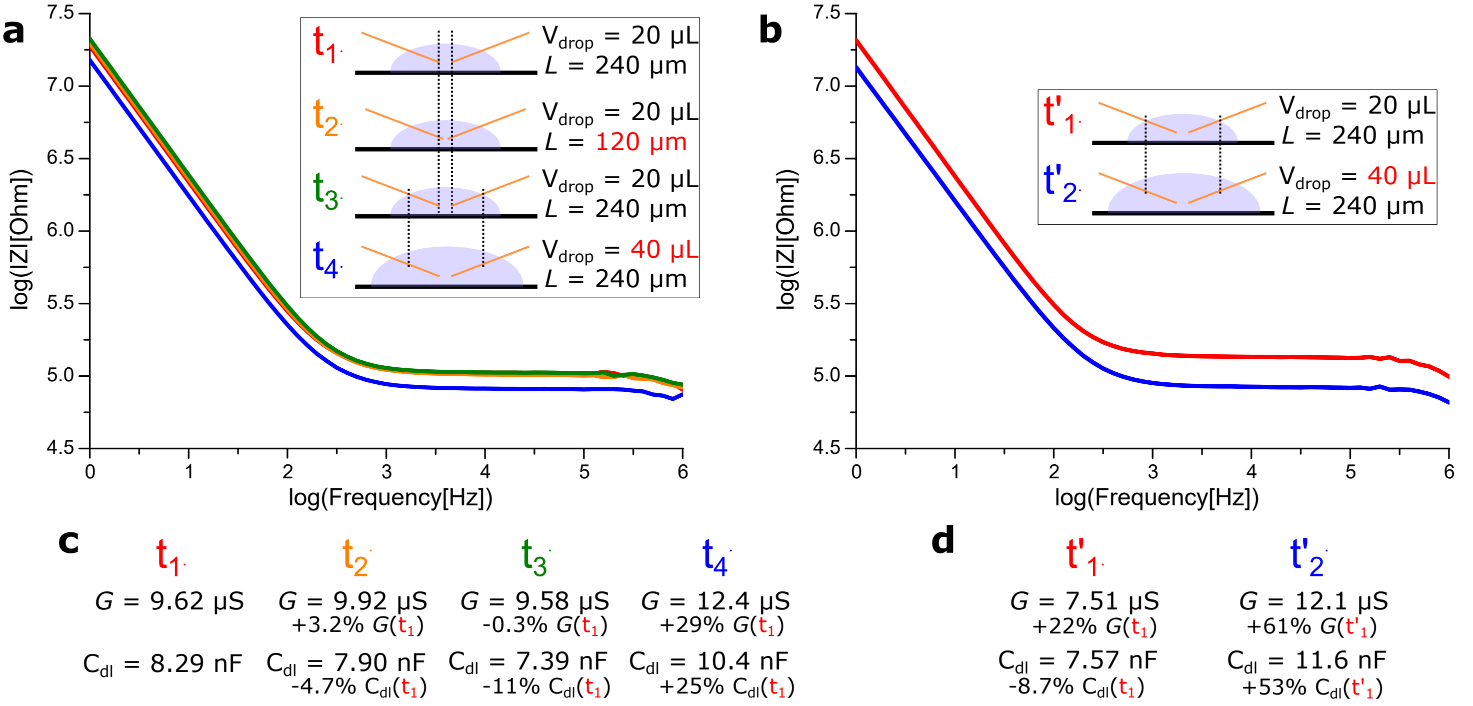

Supplementary Fig. 6 | Stability of the two-wire setup with variation on the drop volume (V_{drop}) and wire gap (*L*). a,b, Impedance spectroscopy for two different setups of two-wires dipped in 1 mM $NaPSS_{(aq)}$ experiencing variations on V_{drop} and *L* (*i* indexes on T_i labels are chronologically sorted). **c,d,** Fitted parameters for the impedance spectra on a serial $1/G+C_{dl}$ circuit model.
